# Rhinophyma

**Published:** 2015-05-01

**Authors:** Jake Laun, Jared Gopman, Joshua B. Elston, Michael A. Harrington

**Affiliations:** Department of Surgery, Division of Plastic Surgery, University of South Florida Morsani College of Medicine, Tampa, FL

**Keywords:** rhinophyma, tangential excision, rosacea, nasal deformity, bulbous

## DESCRIPTION

**Figure 1:**
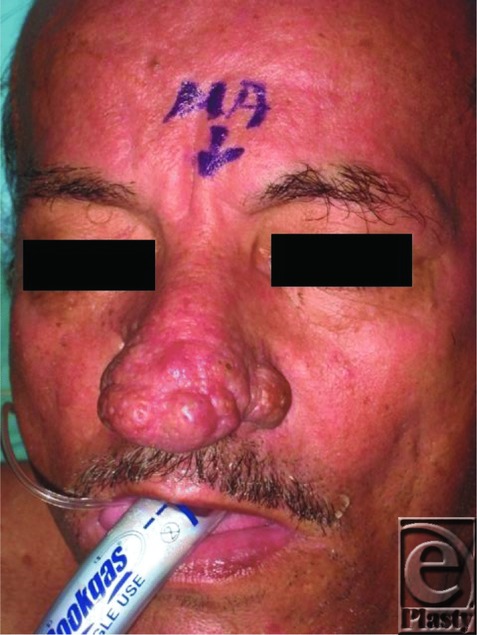
Classic appearance of rhinophyma with a bulbous, cosmetic nasal deformity causing distortion of the aesthetic subunits of the lower nose. Alar thickening can eventually lead to obstruction of the external nasal valves.

**Figure 2:**
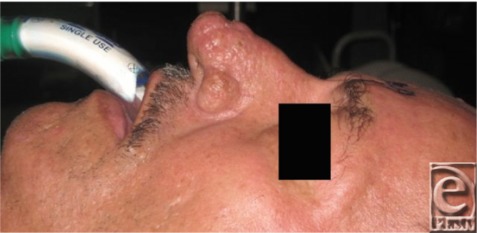
Transition of the affected thicker sebaceous skin of the lower nose and the relatively unaffected upper nasal skin.

A 68-year-old man presented with a large bulbous growth on his lower nose, which he says has been growing for years. He has been embarrassed by the cosmetic appearance for years and relates that he “doesn't even drink that much.” Recently, he has noticed increased difficulty breathing (Figs 1-2).

## QUESTIONS

1. **What is rhinophyma?**
2. **What is the pathogenesis of rhinophyma?**
3. **How is rhinophyma diagnosed?**
4. **What are the main treatment modalities for rhinophyma?**

## DISCUSSION

Rhinophyma (Greek “nose growth”) is a benign skin deformity characterized by tumorous growth leading to a large, bulbous, and erythematous appearing nose.^1^ It is considered to be one characteristic of advanced stage IV rosacea. While rosacea is a common condition found in women, rhinophyma has a male-to-female predominance ranging from 12 to 30:1 and typically occurs in the age range from 50 to 70 years.^2^^-^4 Fortunately, only a minority of patients with rosacea progress to develop rhinophyma. In one study of 108 patients with rosacea, only 15 were noted to have rhinophyma, almost all of whom were men.^5^

The exact cause of rhinophyma is unknown; it is believed to be multifactorial in origin with a principal etiology of unregulated superficial vasodilation.^1^^,^^3^^,^^6^ The extravasation leads to chronic edema of the dermal interstitium with a sequela of local inflammation, fibrosis, and dermal and sebaceous gland hyperplasia.^2^^-^4 Over time, this leads to the characteristic bright red to purplish telangiectasias and irregular, lobulated thickening of the skin of the nose (Fig 1). Historically, rhinophyma was erroneously considered to be linked with alcohol consumption because substances such as alcohol and caffeine can cause local vasodilation, which worsens the symptoms.^2^ This alleged association with alcohol has caused much social stigma and loss of self-esteem in patients suffering from the disease, with several nicknames for the condition such as “whiskey nose” and “rum nose.”^4^

The diagnosis of rhinophyma is clinical and can be identified by the nose's bulbous shape, skin pitting/scarring, and telangiectasias. Most commonly, the thicker and more sebaceous nasal tip and alae are preferentially enlarged, but involvement can spread to the thinner nasal dorsum and sidewalls to a lesser degree (Fig 2). With progression, the aesthetic subunits of the nose merge and become obliterated. While the underlying frameworks are usually unaffected, patients often suffer from secondary nasal airway obstruction at the external nasal valves.^2^^-^4 Rhinophyma can be complicated by unnoticed cutaneous malignancies. Occult basal cell carcinoma is estimated to occur in 3% to 10% of rhinophyma cases, while other types of skin cancers and systemic malignancies have been found to mimic rhinophyma.^2^^-^4

**Figure 3:**
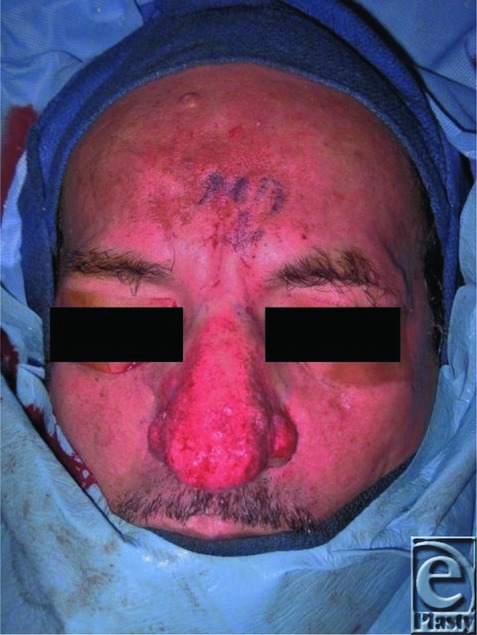
Improved cosmetic appearance after tangential excision. Unaffected areas also resurfaced to give an even final skin tone, rather than a clear transition zone from operated to native skin.

**Figure 4:**
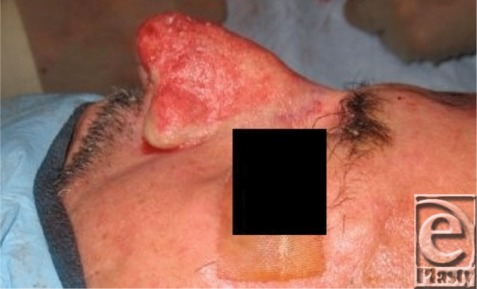
The wound should be kept moist with frequent application of antibiotic cream to promote reepithelialization and to prevent infection.

Although topical antibiotics or retinoids are effective medical treatment options for rosacea, they have not been shown to improve rhinophyma.^2^^,^^3^ Multiple treatment modalities exist for rhinophyma depending on the size of the lesion and experience of the surgeon. Sharp excision, dermabrasion, and cryosurgery are simple, fast, and inexpensive; however, all have varying degrees of poor hemostasis that can obscure the surgical field and lead to uncontrolled depth of tissue removal. Carbon dioxide (CO_2_) laser surgery has become increasingly popular because sharp margins and good hemostasis can be obtained with improved wound healing compared with scalpel excision.^4^ The risk of excessive heating of surrounding tissues leading to scar contracture should be considered and the procedure is cost-intensive and time-consuming.^4^ Success utilizing a triple approach has been reported consisting of tangential excision for debulking, scissors for sculpting, and mild dermabrasion for final contouring (Figs 3-4). In their series of 6 patients, good postoperative hemostasis and improved time to healing was achieved by the triple approach followed by application of an alginate dressing.^7^

Rhinophyma begins as a benign skin condition at the most severe end of the rosacea spectrum with a male predominance. It can lead to severe cosmetic deformities with resulting psychosocial consequences and the possibility of developing occult malignancy. Nonsurgical contouring can yield modest results, but surgical correction appears to be the best for optimal treatment of rhinophyma, with multiple modalities available at the discretion of the surgeon.
